# Down-Regulated miR-30a in Clear Cell Renal Cell Carcinoma Correlated with Tumor Hematogenous Metastasis by Targeting Angiogenesis-Specific DLL4

**DOI:** 10.1371/journal.pone.0067294

**Published:** 2013-06-27

**Authors:** Qing Bo Huang, Xin Ma, Xu Zhang, Shang Wen Liu, Qing Ai, Tao Ping Shi, Yu Zhang, Yu Gao, Yang Fan, Dong Ni, Bao Jun Wang, Hong Zhao Li, Tao Zheng

**Affiliations:** 1 Department of Urology/State Key Laboratory of Kidney Diseases, Chinese PLA General Hospital/PLA Medical School, Beijing, China; 2 Department of Urology, Chinese PLA 303 Hospital, Nanning, China; Queen Elizabeth Hospital, Hong Kong

## Abstract

**Background:**

Endothelial DLL4 plays an important role in controlling of tumor angiogenesis, which is required for tumor invasive growth and metastasis. However, the regulation of DLL4 in clear cell renal cell carcinoma (ccRCC) has not yet been systematically elucidated.

**Methodology:**

We performed bioinformatical analysis to explore miRNAs targeting DLL4. miR-30a was selected as a representative to validate its functional association in endothelial cell. Then, the expressions of DLL4 and mature miR-30a from 90 cases of ccRCC and 28 cases of nonmatched adjacent non-tumor tissues were measured by quantitative real-time PCR. Finally, the expression of miR-30a was correlated with DLL4 expression, tumor features (metastatic condition and microvessel density), and patient metastasis-free survival. The univariate and multivariate analyses were performed to select the risk factors associated with hematogenous metastasis, respectively.

**Principal Findings:**

miR-30a negatively regulated DLL4 and inhibited the proliferation and migration of endothelial cells. DLL4 was up-regulated in ccRCC and further increased in hematogenous metastatic cases, while miR-30a was down-regulated in tumor tissues and further decreased in hematogenous metastatic ccRCC (student t test, all p<0.05). Additionally, expression of miR-30a was inversely correlated with expression of DLL4 and microvessel density (linear correlation analysis, both p<0.05). Low-level miR-30a also indicated a higher probability of developing metastasis (log-rank test, p = 0.010). Most importantly, miR-30a expression was an independent predictor of ccRCC hematogenous metastasis by the univariate analysis and binary logistic regression model (both p<0.05).

**Conclusions:**

Down-regulated miR-30a in ccRCC was associated with tumor hematogenous metastasis through increasing microvessel density by targeting angiogenesis-specific DLL4.

## Introduction

Renal cell carcinoma (RCC) accounts for about 3% of adult malignancies and causes about 90,000 deaths worldwide annually. [1] The most common histologic subtype clear cell renal cell carcinoma (ccRCC) originates in the lining of the proximal renal tubule and resists chemotherapy and radiotherapy. The diagnoses are late and prognoses are poor, because there is no biomarker for the early detection of the malignancy. Thus, about 30% newly diagnosed patients show the evidence of metastases at presentation. [2] In case of metastasis, the response rates of cytokine therapy are about 15%–25% worldwide, and the overall median survival time following progression after cytokine therapy is less than one year [3], [4].

Although the mechanism of metastasis is not fully understood, metastasis is generally accepted to depend on tumor angiogenesis. [5], [6] Recent progress in understanding the biology of ccRCC has led to the identification of vascular endothelial growth factor (VEGF) as a therapeutic target in patients with metastatic ccRCC. VEGF is the most potent proangiogenic protein and leads to increased tumor vasculature and metastatic growth. [7] DLL4, an endothelial ligand of Notch signaling pathway, collaborates with VEGF to initiate important cascades that control tumor angiogenesis and tumor progression. [8], [9] However, in preclinical models, DLL4 attenuation results in the growth inhibition of both VEGF-dependent and VEGF-independent tumors. [10], [11] Therefore, there must be other mechanisms of controlling DLL4.

microRNAs (miRNAs) were shown to negatively regulate gene expression at the post-transcriptional level by binding to the 3′ untranslated region (3′-UTR) of target mRNAs. [12] Recently, miRNAs have been shown to be involved in tumor progression and metastasis in kidney and other cancers. [13]–[15] Accordingly, we searched for miRNAs targeting DLL4 using miRNA target prediction algorithms such as miRDB, TargetScan and PicTar. As a result, miR-30a was found to target DLL4 and rank the first in all these algorithms. However, experimental validation of miR-30a targeting DLL4 has not yet been documented, and the role of miR-30a in ccRCC is yet to be elucidated.

We hypothesized that miRNA-30a might be a new regulator of DLL4 and might play an important role in angiogenesis and tumor progression. To test this hypothesis, we performed luciferase assays to determine whether miR-30a bound to the 3′-UTR of DLL4 and whether miR-30a expression was associated with tumor microvessel density (MVD) and hematogenous metastasis status. We also performed metastasis-free survival analysis to further correlate expression of miR-30a with ccRCC hematogenous metastasis.

## Materials and Methods

### Ethics Statement

Written Informed Consent was obtained from all patients. This study was approved by the Protection of Human Subjects Committee, Chinese People’s Liberation Army (PLA) General Hospital.

### Tissue Samples and Cell Line

A total of 90 cases of ccRCC (Summarized in Table 1) and 28 cases of adjacent non-tumor kidney tissues were obtained from Chinese PLA General Hospital. All ccRCC cases were confirmed by a senior pathologist, and staged based on the 2011 Union for International Cancer Control TNM classification of malignant tumors. Vascular endothelium HUVEC-C cell line was originally acquired from ATCC and maintained in 10 ng/mL VEGF165 (PeproTech, USA).

**Table 1:** The features of the patients and the tumor tissue samples detected.

Variable	No. (%)	Variable	No. (%)
**Gender**		**Grade**	
male	71 (78.9)	1	59 (65.6)
female	19 (21.1)	2	24 (26.7)
**Age (y)**		3	7 (7.8)
≤40	7 (7.8)	**Clinical stage**	
>40, ≤60	57 (63.3)	I	35 (38.9)
>60	26 (28.9)	II	12(13.3)
**BMI**		III	18(20.0)
<25	49(54.4)	IV	25(27.8)
≥25	41 (45.6)	**T stage**	
**Necrosis**		T1	41(45.6)
no	43 (47.8)	T2	18(20.0)
yes	47 (52.2)	T3	26(28.9)
**Tumor size (cm)**		T4	5(5.6)
≤4	21(23.3)	**Metastatic status**	
>4, ≤7	28(31.1)	NM	65(72.2)
>7, ≤10	25(27.8)	LM	6 (6.7)
>10	16 (17.8)	HM	19(21.1)

Abbreviation: BMI: body mass index; NM: tumors involving non-metastasis; LM: tumors involving lymphatic metastases; HM: tumors involving hematogenous metastases.

### Prediction of miRNAs Targeting DLL4

miRNA target predicting algorithms miRDB (http://mirdb.org/miRDB/), TargetScan (http://www.targetscan.org/), and PicTar (http://pictar.mdc-berlin.de/) were used to predict miRNAs targeting DLL4 and the binding regions.

### Transfection and Luciferase Assay

The full-length human DLL4 and its corresponding empty vector PCMV6-XL6 were purchased from the OriGene Company (USA). miR-30a plasmid and its negative control vector pcDNA3.0 were provided by Prof. Guo (Department of Gastroenterology of Chinese PLA General Hospital). miR-30a inhibitor and scramble miRNA were purchased from GenePharma (Shanghai, China). miR-30a inhibitor: 5′-CUU CCA GUC GAG GAU GUU UAC A-3′, scramble miRNA: 5′-CAG UAC UUU UGU GUA GUA CAA-3′. The 3′-UTR of DLL4 mRNA was cloned downstream of a firefly luciferase gene of pMIR-REPORT plasmid (OriGene, USA) to construct the DLL4 3′-UTR vector. pRL-TK plasmid (Promega, USA) was encoded with the renilla luciferase gene. Transfections were performed with Mega-Tran 1.0 transfection reagent (OriGene, USA) as previously described. [16] To perform luciferase assay, 1×10^5^ HUVEC-C cells were transferred to 24-well plates and cultured for 24 hours. Then, miR-30a (0.6 µg) or negative control (0.6 µg) was co-transfected with DLL4 3′UTR plasmid (0.2 µg) and pRL-TK plasmid (0.05 µg) for 48 hours. The Dual-Glo Luciferase Assay System (Promega, USA) was used according to the manufacturer’s instructions. Luminescence was measured on a Centro XS3 LB960 luminometer (Berthold, USA) and firefly activities were normalized to renilla activities for each transfected well. Every experiment was performed in triplicates and repeated 3 times.

### Total RNA Extraction and Quantitative Real-time PCR

Total RNA extraction was performed as previously described. [17] For DLL4, cDNA was synthesized from 0.5 µg to 1 µg of total RNA using a TrueScript 1st Strand cDNA Synthesis Kit for reversing transcriptase-PCR (Aidlab Biotechnologies, China). The amplification was detected using SYBR Green dye (Fermentas, USA). Relative quantification was performed by using the ΔΔCt method, normalizing to TATA box binding protein (TBP) and peptidylprolyl isomerase A (PPIA) mRNA. Specially, for accurately determining DLL4 expression level in microvessels, the relative quantitation of DLL4 expression to microvessel marker CD34 expression was defined as “DLL4 density” in short. The primers used for PCR analysis were reported in Table S1 in Supporting Information. For miR-30a, up to 500 ng of total RNA was reverse transcribed into cDNA using the High Capacity Complementary DNA Reverse Transcription Kit (Applied Biosystems, USA) according to the manufacturer’s instructions. Expression of miR-30a was detected by using TaqMan MiRNA Assays (Applied Biosystems, USA) and normalized to U6.

### Antibodies and Western Blotting

The anti-DLL4 antibody was obtained from Abcam (USA) and the anti-CD34 antibody was obtained from Epitomics (USA). Protein extraction and Western blotting were performed by using standard techniques described previously. [18].

### Cell Proliferation and Migration Analyses

Cell proliferation was analyzed by the MTS assay with the CellTiter 96 Aqueous One Solution (Promega, Madison, WI, USA). Cell migration assay was performed with an 8 µm–pore size membrane Transwell apparatus (Corning Costar Corp., Cambridge, MA, USA). These techniques were carried out as previously described. [17].

### Immunohistochemical Staining for MVD

Formalin-fixed, paraffin-embedded tumor sections that had been confirmed by hematoxylin and eosin staining were deparaffinized and incubated overnight at 4°C with rabbit anti-CD34 antibodies (1∶100 dilution; Epitomics). Secondary detection was carried out with an anti-rabbit peroxidase polymer detection kit (Vector Laboratories). The staining signal was detected with a 3,3′-daiminobenzidine substrate kit (Vector Laboratories). Images were acquired with an Olympus IX81 microscope (Olympus, Japan). The images of tumor sections were taken at 200× magnification or more. MVD was quantified by counting the number of microvessels in five selected fields distributed in four quadrants and the middle of the image.

### Statistical Analysis

The relative quantitation of gene expression detected by real-time PCR was log10 transformed and analyzed by student t test or ANOVA. Linear or rank correlation analysis was performed to determine the correlation between the gene expression levels. The univariate analysis and binary logistic regression model were performed to select the risk factors associated with hematogenous metastasis, respectively. Briefly, all covariables were transformed into binary data, and the tumor metastasis status (hematogenous metastasis or not) was selected as dependent variable. The enter method was used in this model. The Kaplan-Meier method and log-rank test were used to estimate and compare the probability of metastasis-free survival. The significance level was set at p<0.05.

## Results

### miR-30a Targeting DLL4

**Figure 1:**
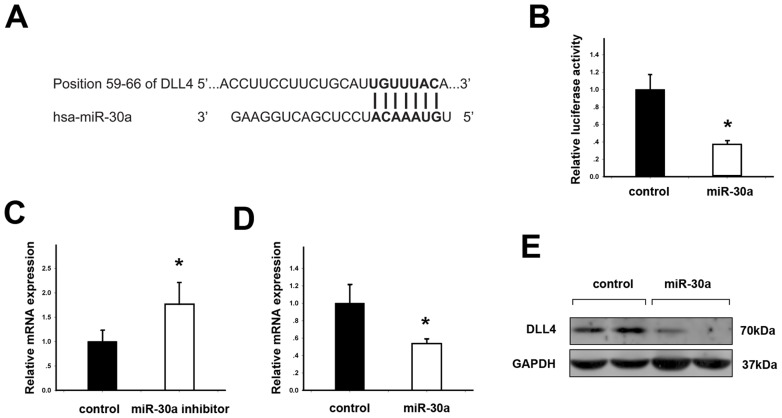
DLL4 is a direct target of miR-30a. (A) Putative complementary site of DLL4 mRNA 3′-UTR for the seed region of miR-30a identified by prediction programs. (B) Endothelial HUVEC-C cells were transfected with DLL4 3′-UTR vector and miR-30a vector or negative control vector (pcDNA3.0) for 48 hours. Relative luciferase activities of DLL4 3′-UTR vector was reduced by miR-30a. (C) HUVEC-C cells were transfected with miR-30a inhibitor or negative control (scramble miRNA) for 24 hours. Relative mRNA expression of DLL4 was increased by miR-30a inhibitor. (D) HUVEC-C cells were transfected with miR-30a or negative control (pcDNA3.0) for 24 hours. Relative mRNA expression of DLL4 was decreased by miR-30a. (E) Western blotting showed that DLL4 was decreased by miR-30a after 48 hours of transfection. Data are represented as the means ± standard errors of the means (SEM). *statistics significant.

To identify the potential miRNAs targeting DLL4, we started with multiple prediction programs and found that miR-30a was one of the miRNAs with the highest possibility of targeting DLL4. The matching consequences of DLL4 3′-UTR and miR-30a predicted by these programs, were shown in Figure 1A. miR-30a expression status was clarified by searching published miRNA profile assays in ccRCC. As a result, miR-30a was found to be down-regulated about two to five fold in different investigations, [19]–[21] which was opposite to up-regulated DLL4 within the vasculature of ccRCC. [22] To validate miR-30a targeting DLL4 experimentally, the luciferase assay was performed. miR-30a transfection suppressed the luciferase activity of DLL4 3′-UTR vector in HUVEC-C cells (Figure 1B). HUVEC-C cells expressed detectable DLL4 under consecutive VEGF stimulation. [23], [24] Thus, real-time PCR and Western blotting were performed to test DLL4 expression when miR-30a was inhibited or ectopically expressed in HUVEC-C cells. DLL4 expression was increased 1.76 fold by miR-30a inhibition (Figure 1C, student t test, p<0.05) and decreased 1.92 fold by miR-30a transfection (Figure 1D and 1E, student t test, p<0.05). Taken together, miR-30a was experimentally validated to target DLL4 in endothelial HUVEC-C cells.

### miR-30a and DLL4 Played Opposite Roles in the Proliferation and Migration of Endothelial Cell

**Figure 2:**
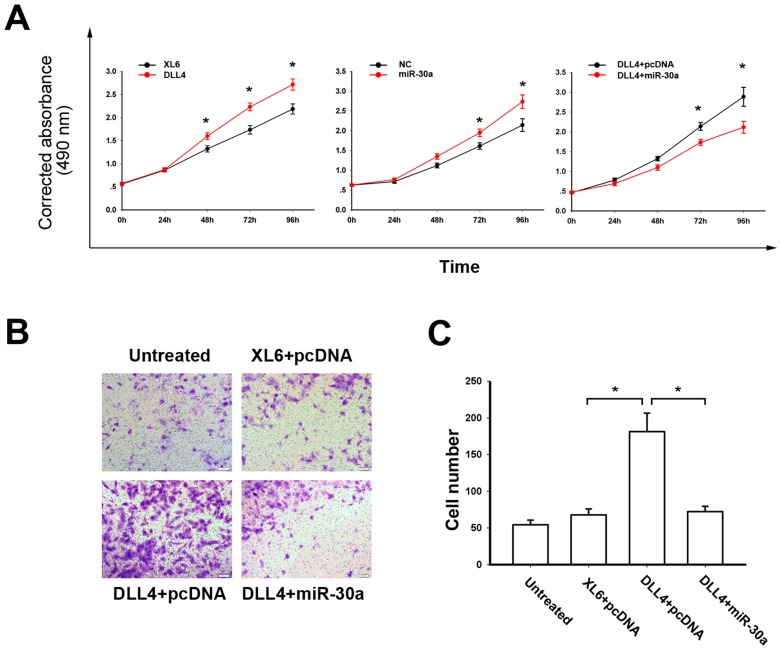
miR-30a and DLL4 played opposite roles in the proliferation and migration of HUVEC-C cells. (A) DLL4-transfected HUVEC-C cells gained more proliferation capacity than control cells detected by MTS assay (left panel), while miR-30a suppressed HUVEC-C cell proliferation relative to control cells (middle panel). HUVEC-C cells were co-transfecting with either miR-30a and DLL4 or DLL4 and pcDNA3.0 and showed that miR-30a partially inhibited the DLL4-stimulated proliferation (right panel). (B) HUVEC-C cells transfected with DLL4 vector and miRNAs control vector (pcDNA) gained more migration capacity than cells transfected with double negative control vectors (XL6+pcDNA). However, HUVEC-C cells transfected with DLL4 vector and miR-30a gained less migration capacity than cells transfected with DLL4 and pcDNA vectors. (C) The numbers of migrated cells in the four groups are shown in bars. Data are presented as the mean ± SEM. *statistics significant.

In the present study, the proliferation capacity of HUVE-C cells detected by the MTS assay was enhanced by overexpressing DLL4 (Figure 2A, left panel). However, over-expression of miR-30a reduced HUVE-C cell proliferation (Figure 2A, middle panel). Then, to determine whether miR-30a affected the proliferation of HUVEC cells stimulated by DLL4, the MTS assay was performed with co-transfecting either miR-30a and DLL4 or DLL4 and pcDNA3.0. As shown in Figure 2A (right panel), miR-30a partially inhibited the DLL4-stimulated proliferation. To determine the roles of DLL4 and miR-30a in endothelial cells, HUVEC-C cells were co-transfected PCMV6-XL6 and pcDNA3.0, or DLL4 and pcDNA3.0, or DLL4 and miR-30a, respectively. As shown in Figure 2B and 2C, miR-30a also partially inhibited the DLL4-stimulated migration. These data suggested that miR-30a targeting DLL4 inhibited the proliferation and migration of endothelial cells.

### High-level DLL4 and Low-level miR-30a were Associated with Higher MVD in ccRCC

**Figure 3:**
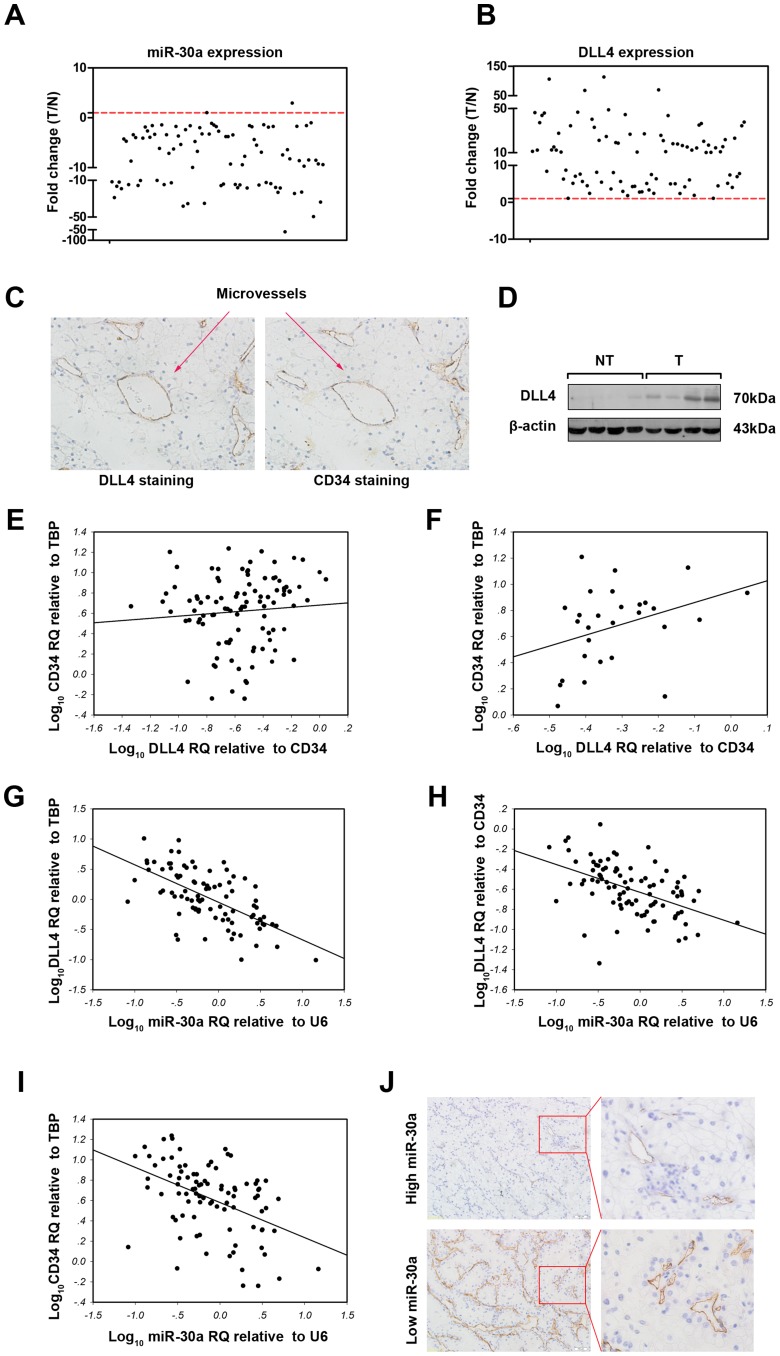
Associations of DLL4 and miR-30a expression with MVD in ccRCC. The expressions of miR-30a (A) and DLL4 (B) in 90 cases of ccRCC and 28 cases of adjacent non-tumor tissues were examined by real-time PCR using U6 and TBP as the internal controls, respectively. Each case of ccRCC was further normalized to the mean levels of 28 cases of adjacent non-tumor tissues. (C) Immunohistochemical staining showed that DLL4 was mainly expressed in the endothelium of ccRCC angiogenesis. (D) Western blotting showed that DLL4 expression was significantly higher in RCC (T) than in adjacent non-tumor tissues (NT). (E) MVD represented by CD34 showed no association with DLL4 density (normalized to CD34, *r* = 0.452, *p* = 0.080). The gene expression levels in each ccRCC sample were log10 transformed before linear correlation analysis was performed. (F) MVD was positively correlated with DLL4 density in about 30% of ccRCC samples (n = 28) with the highest DLL4 density (Spearman correlation analysis, coefficient = 0.388, *p* = 0.042). (G) DLL4 expression was inversely correlated with miR-30a expression in ccRCC (linear correlation analysis, *r* = -0.632, p<0.001). (H) DLL4 density (normalized to CD34) was also inversely correlated with miR-30a expression in ccRCC (linear correlation analysis, *r* = -0.493, *p*<0.001). (I) CD34 expression (MVD) was inversely correlated with miR-30a expression in ccRCC (linear correlation analysis, r = -0.454, p<0.001). (J) miR-30a expression was divided into low or high levels at percentile of 70%. CD34-staining immunohistochemistry showed that less MVD in the high-level miR-30a group than in the low-level miR-30a group.

To date, the regulation of DLL4 by miR-30a in ccRCC has not been well demonstrated. Thus, 90 cases of ccRCC and 28 cases of adjacent non-tumor tissues were collected to examine the expressions of miR-30a and DLL4. Compared with non-tumor tissues, miR-30a expression, detected by real-time PCR, decreased about 10 fold in ccRCC, whereas DLL4 expression was elevated approximate 20 fold (Figures 3A and 3B, student t test, both p<0.05). DLL4 was expressed in tumor endothelium (Figure 3C), and thus the protein level of DLL4 increased in ccRCC (Figure 3D). DLL4 is known to play critical and complex roles in angiogenesis. Therefore, we then determined the association between DLL4 density (normalized to CD34) and MVD represented by the CD34 level. However, no correlation was observed between them (Figure 3E, linear correlation analysis, p>0.05). In light of the idea of Patel et al [22] that an optimal window of DLL4 expression is essential for tumor angiogenesis, we ranked the ccRCC samples according to the DLL4 density level (Figure S1 in Supporting Information). In the about 30% of ccRCC samples (n = 28) with highest DLL4 density, MVD was increased when DLL4 density increased (Figure 3F, spearman rank correlation analysis, p = 0.042). Then, the correlation of miR-30a mRNA expression with DLL4 expression was studied. As shown in Figure 3G, the total DLL4 expression (normalized to TBP) was inversely correlated with miR-30a expression (linear correlation analysis, p<0.001). Importantly, DLL4 density was also inversely correlated with miR-30a (Figure 3H, linear correlation analysis, p<0.001). To further determine whether miR-30a was associated with DLL4-induced angiogenesis in ccRCC, the association between miR-30a expression and MVD was analyzed. As shown in Figure 3I, MVD inversely correlated with miR-30a expression (linear correlation analysis, p<0.001). Furthermore, low level miR-30a associated with higher CD34-staining MVD (Figure 3J). These results indicated that miR-30a down-regulation might induce ccRCC angiogenesis by increasing DLL4.

### Low-level miR-30a was Associated with ccRCC Hematogenous Metastasis and Shorter Metastasis-free Survival

**Figure 4:**
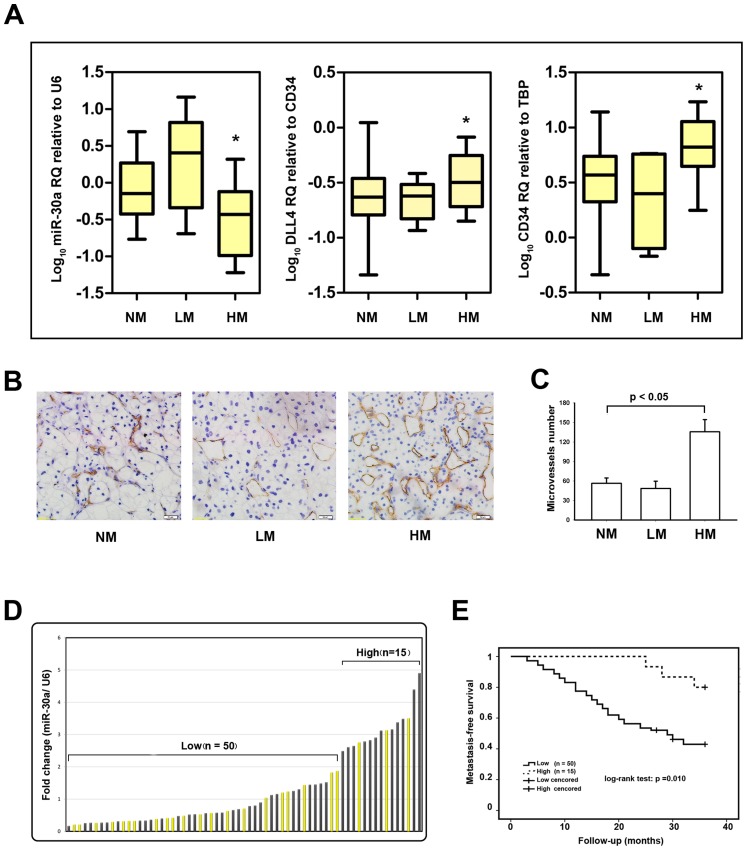
Association of miR-30a with ccRCC hematogenous metastasis and metastasis-free survival. (A) Real-time PCR analyses of miR-30a expression (left panel), DLL4 density (middle panel), and MVD (right panel) in non–metastatic (NM; n = 65), lymphatic metastatic (n = 6) and hematogenous metastatic (HM; n = 19) ccRCC. *p<0.05 based on a comparison between the HM and NM groups. Each box represents the median and 75th and 25th percentile values, and the bars represent minimums and maximums. (B) CD34 staining MVD in ccRCC including NM, LM, HM. (C) Statistical result of MVD in the three groups. (D) A total of 65 cases of non-metastatic RCC were grouped into two, “low miR-30a” (n = 50) and “high miR-30a” (n = 15) according to miR-30a expression at the threshold giving the lowest p value of log-rank test comparing metastasis-free survival between the two groups. (E) Kaplan-Meier graph representing the probability of metastasis-free survival in ccRCC from the two groups. The status was defined as “occurred metastasis or not”, “High” referred to “High-level of miR-30a expression”, while “Low” referred to “Low-level of miR-30a expression”. Log-rank test was used to estimate and compare the probability of metastasis-free survival of the two groups. The *p* value was from a log-rank test.

We then determined whether miR-30a was associated with tumor metastasis. Tumor samples were divided into 3 groups according to the metastatic status. Non-metastatic (NM) samples were obtained from primary sites without lymphatic or distant metastases. Lymphatic metastatic (LM) samples were obtained from primary sites with lymph node metastasis, and hematogenous metastatic (HM) samples were obtained from primary sites in the presence of distant metastases but absence of lymph node metastases. The data showed that miR-30a expression decreased form NM and LM groups to HM group, whereas DLL4 density and MVD increased in HM group. However, there was no statistically significant difference between NM and LM groups in these variables (Figure 4A). Furthermore, CD34-staining immunohistochemistry was carried out in ccRCC and the result validated that MVD in HM group was higher than that in NM or LM groups (Figures 4B and 4C, student t test, p<0.05). These findings indicated that down-regulated miR-30a might increase the risk of hematogenous metastasis by increasing DLL4-induced angiogenesis in ccRCC. To link miR-30a expression to hematogenous metastasis, 65 patients without synchronous metastases were prospectively followed up for a 3-year observational period. These patients were divided into two groups based on relatively high or low levels of miR-30a expression at the threshold giving the lowest p value of log-rank test comparing metastasis-free survival between the two groups (Figures 4D). Metastases developed in 21 out of 50 cases in the low-level miR-30a group, whereas metastasis developed in only 3 out of 15 cases in the high-level miR-30a group during the follow-up period. As shown in Figure 4E, patients in the high-level miR-30a group obtained better metastasis-free survival time than that in the low-level miR-30a group (log-rank test, p = 0.010).

### miR-30a was an Independent Predictor of ccRCC Hematogenous Spread in a Univariate and Multivariate Analyses

For further evaluating the association of miR-30a with hematogenous metastasis, a multivariate logistic regression model was constructed by considering the clinicopathological features. These features involved patients’ characteristics (gender, age, and body mass index), tumor features (size, grade, and pT stage), and miR-30a expression. As shown in table 2, all covariables were transformed into binary data, and the tumor metastasis status (hematogenous metastasis or not) was selected as dependent variable. miR-30a expression was divided into low or high levels at percentile of 70%. This analysis revealed that only miR-30a expression and tumor size were independent predictors of ccRCC hematogenous metastasis. The risk of hematogenous metastasis in tumors expressing low-level miR-30a was 9 fold of that in tumors expressing high-level miR-30a (p = 0.013). In addition, large tumors obtained more possibilities of metastasis than small tumors (odds ratio, OR = 5.776, p = 0.011). In univariable analysis (chi-square test), only miR-30a expression was associated with hematogenous metastasis (p = 0.037). Nevertheless, the tumor size showed marginal statistical significance (p = 0.083). Interestingly, the tumor features, such as grade, and pT stage, were not associated with hematogenous metastasis in this model.

**Table 2:** Univariate and multivariate analyses of hematogenous metastasis- associated factors.

Variables (n)	HM (n, %)	Univariate analysis		Multivariate analyses	
		p	OR	p	OR
**Gender**		0.746	0.431	0.222	0.381
Male (71)	16 (22.5)				
Female (19)	3 (15.8)				
**Age**		0.389	0.717	0.492	1.530
≤60 (64)	12 (18.8)				
>60 (26)	7 (26.9)				
**BMI**		0.168	1.943	0.288	0.507
<25 (49)	13 (26.5)				
≥25 (41)	6 (14.6%)				
**Tumor size**		0.083	3.013	0.011*	5.776
≤7 (49)	7 (14.3)				
>7 (41)	12 (29.3)				
**Grade**		0.182	1.726	0.226	2.189
1 (59)	10 (16.9)				
2&3 (31)	9 (29.0)				
**pT Stage**		0.804	0.061	0.473	0.574
T1& T2 (59)	12 (20.3)				
T3& T4 (31)	7 (22.6)				
**miR-30a expression**		0.037*	5.047	0.013*	0.111
Low (63)	17 (27.0)				
High (27)	2 (7.4)				

Abbreviation: BMI: body mass index; HM: Hematogenous metastasis; OR: odds ratio.

* statistics significant.

## Discussion

Although emerging evidence shows that anti-angiogenesis therapy can partially decrease cancer-specific mortality, the regulation of tumor angiogenesis in mediating cancer metastasis remains unexplored. In the current study, we showed that miR-30a directly targeted DLL4 and decreased tumor MVD, which suppressed ccRCC hematogenous metastasis. Low-level miR-30a was associated with shorter metastasis-free survival. Therefore, early identification of patients at high risk for cancer metastasis by detecting miR-30a expression can identify patients for suitable treatment and may decrease metastasis-specific mortality.

The endothelium-specific Notch ligand DLL4 is well documented to play important roles in embryonic vascular development and tumor angiogenesis. [25] In this study, the results showed that expression of DLL4 was elevated in ccRCC and localized in vasculature. In vitro, DLL4-expressing and VEGF stimulated HUVEC-C cells exhibited increased proliferation and migration, which are important processes in tumor metastasis. Our data confirmed with the findings of Patel et al. [22] However, to our knowledge, DLL4 plays complex roles in angiogenesis. Many reliable studies have reported that the blockage of DLL4 leads to non-productive angiogenesis, [11], [26] which implies that DLL4 inhibits angiogenesis. Similar to the report of Patel et al, optimal window of DLL4 expression is essential for tumor angiogenesis. [22] They found that DLL4 down-regulation produced many phenotypic features displayed by up-regulation of the Notch pathway. In this study, although no significant association was observed between expressions of DLL4 and MVD marker CD34 in all 90 cases of ccRCC, MVD increased with DLL4 density in about 30% of the RCC samples with the highest DLL4 density. Therefore, we propose that tumorigenesis under normal circumstances may be regulated by a complex network involving DLL4 crosstalk with other signaling or factors, for instance, the vasculature-promoting protein VEGF. During tumorigenesis, DLL4 is stimulated by VEGF and expressed in endothelium, but DLL4 controls vessel sprouting and branching triggered by VEGF. [8], [9] The two genes keep angiogenesis balanced with tumor growth under normal circumstances. However, in the present study, we found that high-level DLL4 was associated with high MVD in ccRCC, which was correlated with tumor hematogenous metastasis. This phenomenon challenged that DLL4 played single role in angiogenesis and suggested that it might be regulated by other factors under special circumstance.

miRNAs negatively regulate gene expression by pairing to the target gene mRNAs. [12] When pairing to a target is extensive, as is the common case in plants, miRNAs can direct the destruction of the targeted mRNA through mRNA cleavage. [27] Otherwise, miRNAs repress protein output with little or no influence on the mRNA levels, which is believed to be the dominant mode in animals. [28], [29] The bioinformatical and experimental analyses were performed to explore the involvement of miRNAs targeting DLL4 in endothelium. By searching several prediction programs, we found that miR-30 family had the highest possibility to target DLL4. Recently, Bridge et al showed that miR-30b and miR-30c targeted DLL4 during angiogenesis. [30] In this study, we experimentally validated that miR-30a targeted DLL4 in endothelium for the first time. They played opposite roles in the proliferation and migration of endothelial cells. However, inhibition or forced expression of miR-30a changed both the mRNA and the protein levels of DLL4. In addition, total DLL4 mRNA expression and DLL4 density were inversely correlated with miR-30a in ccRCC samples. These findings were inconsistent with the common recognition that miRNAs repress the protein output with little or no influence on mRNA levels in animals. Nevertheless, Guo el al. [31] used ribosome profiling and found that mammalian miRNAs predominantly acted to decrease target mRNA levels and that lowered mRNA levels accounted for most (≥84%) of decrease in protein production. In addition, several mRNA-array experiments have shown that miRNAs decreased the levels of many targeted mRNAs. [32]–[34] These results support our findings.

The widespread and comprehensive use of microRNA microarrays has enabled the identification cancer-specific microRNAs as potential biomarkers for diagnosis, prognosis and targets for therapy. [35] Recently, several profiling analyses reported that miRNAs were involved in tumorigenesis and metastasis in RCC. [13], [21], [36] Interestingly, miR-30 family members were found to play critical role in pronephros development or apoptosis in renal cell carcinoma. [37], [38] Moreover, miR-30 family members were down-regulated in ccRCC and further down-regulated in metastatic RCC the current work and previous ones. [13], [19], [20], [39] Previous studies have shown that miRNAs were specifically involved in the critical steps of metastatic cascades, for example, tumor angiogenesis. [40]–[42] In order to develop in size and metastatic potential, the tumor must undergo an “angiogenic switch”, which provides a rich blood supply and sheds tumor cells from the primary tumor to distant sites. [6] The results of this study showed that down-regulation of miR-30a was correlated with high MVD in ccRCC, which was further associated with ccRCC hematogenous metastasis. During a 3-year observational follow-up, the low-level miR-30a indicated shorter metastasis-free survival time. Additionally, the univariate and multivariate analysis demonstrated that miR-30a was an independent predictor of ccRCC hematogenous spread. Given that angiogenesis-specific DLL4 has been proven to be a target of miR-30a, we propose that miR-30a down-regulation may promote ccRCC hematogenous metastasis through increasing MVD by targeting angiogenesis-specific DLL4. Nevertheless, further studies are needed to confirm that miR-30a is down-regulated in both endothelium and tumor cells because recent investigations have shown that microvesicles released from tumor cells can act as mediators of intercellular communications. [43]–[45] These microvesicles contain miRNAs and enable miRNAs from tumor or stromal cells to target endothelium specific factors. As expected, miR-30a was found to be down-regulated in microvesicles released from RCC. [41] In the present study, DLL4 mRNA expression was inversely correlated with miR-30a expression, which further supported our conclusion.

In summary, miR-30a was down-regulated in RCC and further decreased in RCC with hematogenous metastasis. Mechanistically, down-regulation of miR-30a increased microvessel density by targeting DLL4. Clinically, low-level miR-30a indicated a higher probability of developing metastasis and shorter metastasis-free survival.

## Supporting Information

Figure S1 **The association of DLL4 density with microvessel density.**
(DOC)

Table S1 **Real-time RT-PCR Primers.**
(DOC)
