# Supplementary material for: Down-Regulated miR-30a in Clear Cell Renal Cell Carcinoma Correlated with Tumor Hematogenous Metastasis by Targeting Angiogenesis-Specific DLL4
Source: PLoS One. 2013 Jun 27;8(6):e67294. doi: 10.1371/journal.pone.0067294 (PMC3694928; doi:10.1371/journal.pone.0067294)
Supplement: Figure S1 — The association of DLL4 density with microvessel density. (DOC) [file pone.0067294.s001.doc]

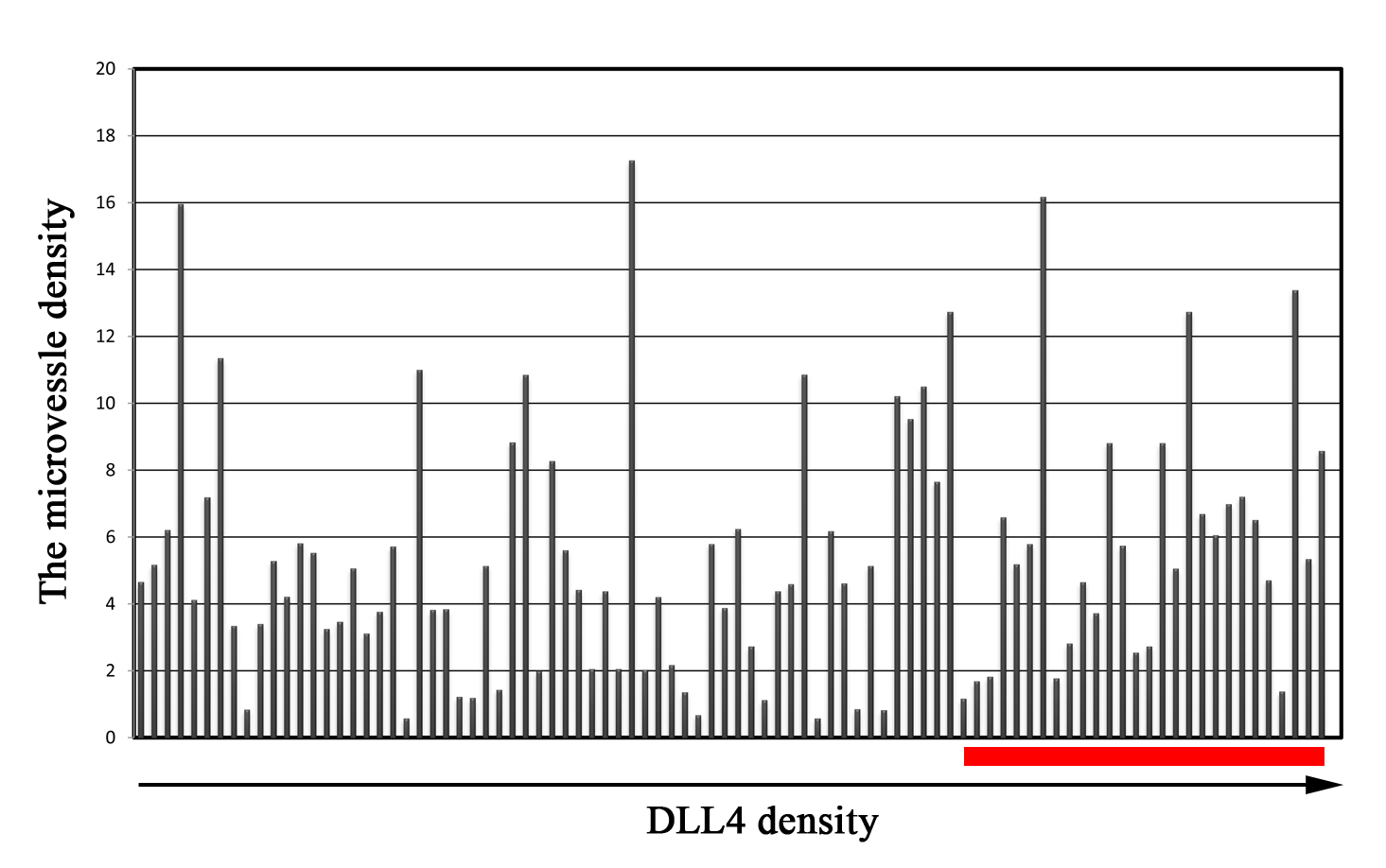


Legend

Figure S1. The association of DLL4 density with microvessel density. The samples were ranked by DLL4 density, and the microvessel density level was represented by the black bars. The samples above the red bar represented the about 30% of ccRCC samples (n=28) with highest DLL4 density.
