# Supplementary material for: Down-Regulated miR-30a in Clear Cell Renal Cell Carcinoma Correlated with Tumor Hematogenous Metastasis by Targeting Angiogenesis-Specific DLL4
Source: PLoS One. 2013 Jun 27;8(6):e67294. doi: 10.1371/journal.pone.0067294 (PMC3694928; doi:10.1371/journal.pone.0067294)
Supplement: Table S1 — Real-time RT-PCR Primers. (DOC) [file pone.0067294.s002.doc]

Table S1. Real-time RT-PCR Primers

| Target Genes | Sense | Antisense |
| --- | --- | --- |
| DLL4 | CTGCGGTTACACAGTGAA | ACATTCATTCCTCTCCTCTG |
| CD34 | TCTCCCACTAAACCCTATACA | CTTCTCTGATGCCTGAACAT |
| TBP | CACGAACCACGGCACTGATT | TTTTCTTGCTGCCAGTCTGGAC |
| PPIA | TCATCTGCACTGCCAAGACTG | CATGCCTTCTTTCACTTTGCC |
